# The Tuberculin Test in Cattle

**Published:** 1901-09

**Authors:** G. S. Pollard

**Affiliations:** Midsomer-Norton.


### THE TUBERCULIN TEST IN CATTLE.

BY

G. S. Pollard, L.R.C.P., M.R.C.S., Midsomer-Norton.

Last March, as Vice-Chairman of the North-east Somerset Farmers' Club, I read a paper on "Tuberculosis in Cattle." A large number of the members were present, and entered heartily into the debate held afterwards. As a result of that paper, I am glad to say that several farmers have had their cattle inoculated.

I pointed out to them that all domestic animals are more or less subject to tuberculosis—although in some only by inoculation. Cattle come first among its victims, the proportion attacked varying according to the locality (in some places from 10 per cent. to—in Cheshire—70 per cent.) Horses may be infected under the ordinary condition of their keeping. Sheep and goats are remarkably free. In the pig tuberculosis is rarer than in cattle, but more common than in the horse.

I now wish to draw attention:—

1st. To the ordinary symptoms of tuberculosis in the cow; and the value of the tuberculin test, with the temperature chart showing the results of the test in one dairy.

2nd. To some points regarding the milk supply.

Pulmonary tuberculosis is much the most prevalent form.

In the *first* stage a cough may be noticed at intervals, or when the animal is made to stand up or walk, although there may be no general symptoms.

In the *second* stage the disease is more pronounced. The hair loses its gloss, and becomes dull and bristly; the skin is adherent and dry, and, if taken between the fingers, is with difficulty detached from the subjacent tissues. The cough is dry, although at times mucus may be shot forth from the mouth. Generally, however, this mucus is swallowed; and one can easily watch the bolus in its course. The milk is less in quantity, slightly serous, and has a bluish tint.

In the *last* stage the wasting is more marked; the skin is bound down to the bones, and the eyes are watery and sink back in their orbits.

A few cows in this condition are a great source of danger to the other members of the herd—others being infected by inhaling the breath, and through the food—the virulent expectoration being ejected from the mouth during fits of coughing, and thereafter swallowed by other cows. Some of the expectorated matter becomes dry and adheres to the manger, and may be licked off by another animal.

The most dangerous condition to the human subject is tuberculosis of the udder. According to Bang and Bollinger, the bacilli are abundant in the milk taken from such udders. For a month or so after the disease has commenced, the milk is apparently unaltered, even although it may prove to be highly contagious. The milk subsequently becomes thin and serous.

Some German pathologists have rendered pigs tubercular within five or six days by feeding them on milk taken from a tuberculous cow. Quite recently, of nine pigs killed locally, seven were found to have tuberculosis. Bang states that the tuberculous udder is not so rare as might be supposed. It presents itself as a painless tumour found in one or other quarter of the gland; the large size of the udder, its unusual hardness, and the complete absence of signs of suppuration, are distinctive diagnostic features.

The tuberculin test is of such value that all dairy animals should be subjected to it. This test consists of a glycerine extract of a pure cultivation of tubercle bacilli; and when injected under the skin of cattle suspected of tuberculosis, it sets up (in tuberculous animals alone) a febrile reaction, permitting one to assert the existence of even minute lesions. Nocard states that the injection of a strong dose, say 30 to 40 centigrams, according to the size of the animal, generally causes in tuberculous animals a rise of temperature between 1½ to 3 degrees. In healthy cattle, the same dose causes no appreciable febrile action. In very tuberculous animals, especially in those with fever, the reaction may be little marked, or even *nil*.

Nocard recommends that the temperature be taken night and morning for several days before the injection, as the animal may be suffering from some other ailment. The tuberculous calf reacts just as well as the tuberculous adult. The injections of tuberculin have no troublesome effect on the quality or quantity of the milk or on the progress of gestation.

The temperature chart gives the record of temperatures taken night and morning, on the day before inoculation in the first and second columns. Some of the cattle had a slight rise of temperature, especially at night. This is most marked in No. 20 (Bull). The following three columns show the result after the animals had been inoculated, and in fourteen cases show a decided rise of temperature due to the test, which was applied immediately after the taking of the temperature on the previous evening. The syringe had been boiled, and the skin over the dewlap was washed with 1 in 20 carbolic lotion. The first temperature was taken at 6 a.m. (ten hours after inoculation), and again at 9 and 12 a.m., being thirteen and sixteen hours after injection. A few cases are doubtful, and will be therefore again subjected to the test, or sold off the farm. This test must not be repeated within six weeks or more, for it has been known for some time that a first reaction in a tuberculous animal may prevent one from obtaining a second decided reaction when the test is repeated too soon afterwards.

The Report of the Tuberculin Committee of the Royal

Agricultural Society of England sums up the results of their experiments as follows:—

1. With few exceptions, manifest tuberculous disease is discoverable at the *post-mortem* examination of animals in which there is a decided rise of temperature after the injection of tuberculin.
2. As a rule, no such lesions are to be found in those animals in which there is no decided rise of temperature after the injection of tuberculin; but the exceptions to this rule are more numerous than in the preceding case.

My list of twenty-nine cases does not represent all the animals inoculated in the district. I have been privately informed that the farmers are having their cattle inoculated: some, only for their own use; others, to enable them to sell all doubtful cattle off their farms. On one farm, twenty cows were inoculated, and two only found to be tuberculous; this must have been an exceptionally fine lot of stock.

As regards the number of tuberculous animals:—

In *1891* 10,000 animals in different parts of the British Isles were slaughtered for pleuro-pneumonia, or for having been in contact with it. *Post-mortems* were made on these 10,000 animals, and 1,260 were tuberculous, or 12½ per cent.

In *1892*, for the same cause, 3,600 were slaughtered, and 800 were tuberculous, or 22 per cent.

Mr. S. S. Cameron, Veterinary Inspector for the Board of Health in Victoria, states, in his Report on the use of tuberculin in cattle, that 267 cattle were tested: of these, 23 gave the result, and were slaughtered. *Post-mortem* examinations were made in every case, and tubercles were found in all. In 21 per cent. of these animals slaughtered, tuberculosis of the udder was found.

A writer in the *British Medical Journal*[1] remarks as follows: "It is now admitted that in all probability some 70 per cent. of the milch cows in this country are more or less tuberculous."

Mr. Haydon, of Midsomer-Norton, has tested 300 young stock, and found that 5 per cent. reacted to the tuberculin test.

[1] 1899, i. 1355.

Now as regards the milk supply. Is the milk that we obtain to-day the natural product of the cow? I do not think it is. I should rather call it the product of an over-stimulated udder, obtained by high and over feeding. To obtain a large milk supply from the dairy, it is necessary for the farmer to carry out the following system:—

1st. *Warmth.*—The cowshed must be kept at a high temperature; and to obtain this, the animals are kept as close together as possible, and free ventilation is prevented. This assists the spread of tuberculosis, the animals being kept in the shed day and night.

2nd. *Feeding.*—It is necessary to be very careful about the feeding. A certain amount of hay is given; large quantities of brewers' grain; cotton and oil cake, mangel and ensilage. Some farmers state that the latter (ensilage) increases the milk supply by 15 per cent. This enables the farmer to obtain from one quart to one gallon extra per cow. The selling price of milk is 8d. per gallon in winter and 4d. per gallon in summer, and the farmer has to pay the railway carriage out of this. The result of this high feeding, leaving out the danger of tuberculosis which we medical men see, is the serious danger to infant life from other causes. Brewers' grains quickly cause milk to ferment, and thus cause infantile diarrhœa. Milk from cows heavily caked will soon upset children; stop the cake, and the children are soon well. Hay in excess soon makes milk poor, and causes it to lose its anti-scorbutic properties, so that the children get rickets. This anti-scorbutic action of milk is one of its most valuable properties, but it is lost by boiling, and I am very doubtful if pasteurising does not do the same. I find that children fed on boiled milk are anæmic and rickety. Some that are fed on pasteurised milk are certainly anæmic.

I have also attended children with diarrhœa, who have been fed on milk from selected cows. In each case, I have traced the complaint to a change of diet in the cow, and have only cured the child by stopping the milk and giving it chicken broth.

I always advise my patients, who can afford it, to keep a cow of their own; to use the tuberculin test; and to keep the cow at pasture all the year—in winter by day, and giving it, in addition, hay, corn, and cabbage; so keeping the animal in health, and avoiding the product of an over-stimulated udder.

One result produced by high feeding for the supply of winter milk is the number of cows and heifers which are drafted off each year for grazing; and this is, I am sorry to say, steadily increasing. A large percentage of these animals have tuberculosis. If this class of animal is turned out into good pastures and given a certain amount of cake, they soon become fat and fit for the butcher. For the last few years I have frequently visited slaughter-houses, and have found that 50 per cent. of cows and heifers killed have had tuberculosis. Some of them before slaughter have been magnificent animals to look at, but have been simply studded with tuberculosis, both pulmonary and abdominal.

Having tested our dairy, what are we to do with the tuberculous animal? Slaughtering is not possible, as the cost would be enormous. The total number of cattle in England is nearly seven millions, and on June 4th, 1900, the number of cows and heifers in milk or in calf was 2,620,901. Only allowing 10 per cent. to have tuberculosis, the slaughtering would cost £3,000,000. So we see that slaughtering with or without compensation is impossible. What would it cost if the *British Medical Journal* with its 70 per cent. is correct?[1]

Bang states that a healthy stock may be bred from a tuberculous herd by the simple precaution of separating the healthy from the infected animals, and boiling the milk of tuberculous cows. By doing this, one could in five years have a healthy herd on every farm. A farmer who was present at my lecture in March confirmed this statement, saying that he had been on the Continent and seen Bang's method of separating the healthy cattle, and taking the calves away from their tubercular mothers at birth.

To summarise our points:—

1st. The value of the tuberculin test is incalculable.

2nd. The danger of tuberculous milk, especially when the udder is affected, is very great.

[1] *Loc. cit.*

### TEMPERATURE CHART.

| No. | THURSDAY. | | FRIDAY. | | | |
|---|---|---|---|---|---|---|
| | 9.0 a.m. | 8.0 p.m. | 6.0 a.m. | 9.0 a.m. | 12.0 a.m. | |
| 1. Heifer ... ... | 101.4 | 101.8 | 101 | 101 | 102 | |
| 2. Heifer ... .. | 101.4 | 102.2 | 100.8 | 101 | 101 | |
| 3. Heifer ... ... | **102.2** | **102.2** | **105** | **103** | **104.2** | Tuberculous. |
| 4. Heifer ... ... | **102.2** | **103** | **105** | **104.8** | **104** | Tuberculous. |
| 5. Heifer ... ... | 102 | 102 | 103 | 103 | 103.2 | |
| 6. Cow ... ... | 101.4 | 101.8 | 101 | 103.4 | 102.2 | |
| 7. Cow ... ... | 101.2 | 101.6 | 101 | 101.2 | 101 | |
| 8. Cow ... ... | 100.8 | 102.2 | 100.2 | 101 | 102 | |
| 9. Cow ... ... | 101.6 | 102.2 | 100.6 | 102 | 102.6 | |
| 10. Cow ... ... | 101 | 101.8 | 100.8 | 100 | 100 | |
| 11. Cow, *coughs and grunts* | **100.8** | **102** | **104.8** | **105.2** | **106** | Tuberculous. |
| 12. Cow ... ... | **101.2** | **101.8** | **102** | **104.8** | **104.2** | Tuberculous. |
| 13. Cow ... ... | 101 | 101.6 | 101 | 101 | 101 | |
| 14. Cow ... ... | **101** | **102.2** | **103** | **105.4** | **103.6** | Tuberculous. |
| 15. Cow ... .. | 101 | 102.4 | 101 | 101 | 102 | |
| 16. Cow, *coughs and grunts* | **101.4** | **103** | **105.4** | **105.4** | **106** | Tuberculous. |
| 17. Cow ... ... | **101.2** | **101.6** | **103** | **104** | **103.8** | Tuberculous. |
| 18. Cow ... ... | **101.4** | **102** | **100.8** | **105** | **100.4** | Doubtful. |
| 19. Cow ... ... | **101.2** | 102.2 | **105.2** | **105** | **104** | Tuberculous. |
| 20. Bull ... ... | **102.4** | **103** | **106** | **106.2** | **106** | Tuberculous. |
| 21. Cow ... .. | **101** | **102** | **104** | **105** | **104** | Tuberculous. |
| 22. Cow ... ... | 100.8 | 102 | 100.8 | 101 | 101 | |
| 23. Cow ... ... | 101.4 | 101.8 | 101 | 100.4 | 100.4 | |
| 24. Cow ... ... | **100** | **101.6** | **100** | **101** | **105.8** | Doubtful. |
| 25. Cow ... ... | **101** | **101.2** | **104.8** | **104** | **104** | Tuberculous. |
| 26. Cow ... ... | 101 | 101.6 | 101 | 101 | 101 | |
| 27. Cow ... ... | **101** | **102.4** | **105** | **104** | **104.8** | Tuberculous. |
| 28. Cow ... ... | 101 | 101.4 | 100.6 | 101 | 101 | |
| 29. Cow ... ... | 101 | 102 | 100 | 100.2 | 101 | |

Columns I. and II. taken on the day before, and Columns III., IV., and V. show any decided rise in temperature after inoculation with Tuberculin. In tuberculous cases the rise of temperature is maintained. In those marked doubtful the temperature varies.

3rd. Healthy cattle can be raised from tuberculous mothers.

4th. On account of over-production of milk, more animals become tubercular, and are converted into beef. Public slaughter-houses under proper supervision are urgently required.

[We appear to be much behind the European Continent as regards the use of the tuberculin test. The Editor observed, only a week or two since, in a large hotel in central France there was posted on the walls a certificate of a local veterinary surgeon giving a record of the temperature of the cows belonging to the hotel for a few days after injection of tuberculin.—Ed.]

---

